# Intelligence

**Published:** 1831-04

**Authors:** 


### INTELLIGENCE.

### *Admission of* Naval Surgeons *to the* King's Levees.

A deputation from the surgeons of London waited upon the Duke of Devonshire, on the 8th instant, on the subject of the exclusion of the surgeons and assistant surgeons of the navy from his Majesty's levees. His Grace authorised the deputation to communicate the following answer: "That his Majesty entertained the kindest feelings towards the surgeons and assistant surgeons of his navy; that the order complained of was rescinded; and that, in future, these officers would be admitted to the levees, through the lords of the Admiralty."

### *Power of the* Company of Apothecaries *over the* Members *of the* College of Surgeons.

It has recently been decided, in an action at law, at the Maidstone assizes, brought by the Apothecaries' Company, against a surgeon of the name of Ryan,—that a member of the College of Surgeons cannot legally dispense medicines to his patients, unless he be an admitted apothecary, or unless he was in actual practice as an apothecary before the 1st of August, 1815.

### HUNTERIAN SOCIETY.

*Prize Medal.* The subject appointed by the Council for the gold Hunterian medal, of ten guineas value, is "Unnatural Growths and Deposits of Bone." Dissertations must be sent to the secretaries, on or before the 14th of December, 1831. They must not be written in the hand of the author, and his name must be contained in a sealed packet: it is not required that he be a member of the Society. Unsuccessful papers will be returned, if required.

### LITERARY NOTICES.

Baron Heurteloup has a work in the press, on the "Principles of Lithotrity, or a Treatise on the Art of Curing the Stone without Incision."

Dr. Uwins is preparing for the press a "Treatise on Mental Derangement," in which the subject of insanity will be considered in all its bearings, statistical, pathological, preventive, and curative. In this work, Dr. U. will treat generally on nervous ailments, and their connexion with disorders of the stomach and other organs.

### BIOGRAPHY

### *Of the late* Mr. Robert Chessher, *of Hinckley.*

If unweared industry, joined with an ardent desire to improve the healing art, in any of its branches, deserve notice, then the simple record here given of one who had these qualities in their amplest signification, claims the attention of the profession at large.

Mr. Chessher was the son of respectable parents, who resided for many years in Castle street, Hinckley. After the death of his father, the widow married a Mr. Whalley, surgeon-apothecary of the same place, and with whom Mr. Chessher, the subject of this memorial, served his apprenticeship.

The stepfather was exceedingly kind to his pupil, and often expressed great desire that he should excel in the profession in which he was now initiated. Indeed, this disposition of friendly interest Mr. Whalley realized at his death, by leaving to his son in law the entire control of a handsome property. Mr. Chessher was thus enabled to go through the routine of lectures in London, and he was for some time house-surgeon to the Middlesex Hospital; from the advantages of which situation we may date the extensive practical knowledge he possessed.

It is now near fifty years since Mr. Chessher settled in his native place as a general practitioner: possessing a better knowledge of surgery than the generality of country practitioners then did, (or, at least, those in the immediate neighbourhood,) he obtained a respectable practice; and, during these several years' practice, he performed many of the great operations with success, and was considered as a man well acquainted with anatomy. Hence, in serious or doubtful cases, his opinion was generally required, and he obtained a well-deserved popularity.

After several years thus spent, through the advice of some professional friends, he was disposed to quit his general practice, and to give his attention to diseases of the spine and to deformities of the extremities. In this he was strongly urged by the celebrated Dr. Denman, to whom he had been a private pupil, and with whom he had the honour and enjoyment of many years' friendship and frequent intercourse.*

Through Dr. Denman's instrumentality, Mr. C. obtained patients of the first families, in most of whose cases he was so far fortunately successful, that from these the practice continued to increase for many years, so much so, that Mr. C. has been known to have no less than four hundred patients under his care at the same time; and, to his credit be it be spoken, where his peculiar practice did not succeed, the patients were still satisfied both with his professional skill, and with what had been attempted as a cure.

In Nichol's "History of Hinckley," (a writer of no mean authority,) will be found the following notice of this celebrated practitioner:

"It is impossible to pass over the town of Hinckley, without paying some attention to the uncommon merits of Mr. Robert Chessher, surgeon, who is a native and resident of this place. Having been regularly educated to the profession, he acquired great eminence in the general practice of it; but, many years ago, he relinquished all the other branches, except upon extraordinary occasions, and dedicated his time and abilities to the discovery or improvement of that art, by which young people, distorted in their persons, or those who, more advanced in age, were by various accidents crippled in their limbs, might be restored to their proper shape, or their wonted strength and agility. For this pursuit he was particularly well qualified by his skill in anatomy, and his talent for mechanics, and he has certainly carried it to a degree of perfection hitherto unknown. In consequence of the great reputation he has gained, the town of Hinckley is much resorted to, by patients from every part of his Majesty's dominions, and many foreign countries. His success has, in many of these cases, been astonishing; and it is but justice to mention Mr. Chessher's great liberality in accommodating his charges to the circumstances of his patients, so that even the poor partake of the benefits of his practice and experience."†

During the time that Mr. Chessher was at the zenith of his popularity, he was offered, but declined, the honour of knighthood.

He was a man of very active turn of mind, and this disposition continued to his latest days. It is well known he spent the greater part of his time in his professional avocations: even at a very advanced age, and during the intervals of relaxation from bodily exercise, still did he evince an earnest desire to be in-

* The obligations rendered by this individual, an ornament to his profession, have been noticed by Mr. C., in a handsome bequest to the family.

† Folio edition, printed 1813.—A similar account to the foregoing may be noticed in the Gentleman's Magazine for January 1810, in which a comparison is drawn between the celebrated Venel and the subject of this memorial.

formed of what was passing in the medical world. In the periodicals of the day read by himself, may be seen his marginal notes on subjects of interest, particularly such as concerned his branch of practice: in an original paper of the London Medical and Physical Journal, is a well-described case, by Dr. Griffin, on Functional Disorders of the Spinal Cord,* and a case of Disease of the Hip-joint, by Mr. Thomas, of Liverpool.† These shew how carefully he must have read them, and are given as an instance of what the man was late in life. Hence we may fairly argue that, in his early days, his energy of mind was proportionally great.

It is but a few years since the double-inclined plane for fractures of the thigh was recommended by a great surgical authority, and that gentleman has now the credit of being the inventor and the promoter of its general adoption in these cases. Among many other improvements in machinery and surgical instruments, there will be found, in Mr. Chessher's collection, (now in the possession of Mr. C. Ridley,) the model of this double-inclined plane, which was shewn to the heads of the profession in London upwards of forty years ago. This improvement Mr. C. has been heard frequently to assert is his own idea: it was often used by himself during the time of his being a general practitioner, and he found it to be of essential service. This is not the only surgical improvement for which we are indebted to this indefatigable individual, for the invention of which others assume to themselves the merit.

Mr. C. possessed many anatomical preparations illustrative of the various degrees of distortions, with others relating to surgery in general, the whole forming a valuable collection: these Mr. C. Ridley is now so fortunate as to possess, by the bequest of the late Mr. Chessher.

It would be easy to adduce quotations from writers of distinction, to shew that Mr. Chessher's practice was in accordance with the opinions of many of the first authority; and with those who strongly objected to the use of machinery, his method was said to be least objectionable, or, if any mechanical contrivance did good, it was that of Mr. Chessher.

Speaking generally of Mr. Chessher's practice, it may be noticed, that it was never pursued in one unvaried manner, but the treatment in each case was according to its peculiar features; thus comprising, in the whole, the many popular methods of late followed by others who have acquired notoriety: yet, in fairness to Mr. Chessher, we would here remark, that the violent and unjustifiable measures used by some, by no means formed a part of his treatment; the necessity of attending to the general health, he always kept in view. In spinal diseases, we have too often unfortunate proof of the difficulty of curing or alleviating them by means of art: at the same time, we have frequent opportunities of witnessing the great efforts of nature to surmount the effects of these terrible diseases.

Many men of eminence object to the use of machinery altogether:‡ their authority individually is to be attended to, and collectively is of great importance; yet some of these, and men high in the world's opinion, have argued most inconsistently, by decrying the use of supports, whilst they still recommend the same themselves, varying only the form, without taking advantage of the nicety of mechanical adaptation.§ The advantages of mechanism Mr. C. possessed, by having about him men of unquestionable skill in that department, and whose whole attention and employment were directed to every variety of instrument used in cases of deformity. Here it is but justice to notice the valuable services of Mr. John Felton, of Hinckley: his mechanical skill and experience, for many years, have mainly contributed to the success of machinery in many of the most serious cases.

* December 1829, p. 487. † Ibid. p. 491.

‡ See Arnott's "Elements of Physics," second edition, p. 199 to 205.

§ See Shaw on "Distortion of the Spine."

After a protracted illness of many months, by a gradual decay of nature, Mr. Chessher died on the 31st January last, at the advanced age of seventy-nine years.

The peculiar mode of practice for so many years followed with success by Mr. C., is now pursued by Mr. Charles Ridley, surgeon, an enterprising and deserving young man.

### MONTHLY LIST OF MEDICAL BOOKS.

### METEOROLOGICAL JOURNAL,

By Messrs. Harris and Co. Mathematical Instrument Makers, 50, High Holborn.

| | Moon. | Rain gauge. | Thermom. | | | Barometer. | | De Luc's Hygrometer. | | Winds. | | Atmospheric Variation. | | |
|---|---|---|---|---|---|---|---|---|---|---|---|---|---|---|
| Feb. | | | 9 a.m. | max. | min. | 9 a.m. | 10 p.m. | 9 a.m. | 10 p.m. | 9 a.m. | 10 p.m. | 9 a.m. | 2 p.m. | 10 p.m. |
| 20 | | | 47 | 48 | 41 | 29.91 | 29.90 | 63 | 61 | W | NW | Cloudy | Rain | Fine |
| 21 | | .45 | 42 | 43 | 34 | .86 | .82 | 60 | 60 | NW | N | Fine | Fine | — |
| 22 | | | 40 | 43 | 37 | .89 | .90 | 58 | 59 | N | NNW | — | — | — |
| 23 | | | 39 | 40 | 35 | .90 | 30.13 | 59 | 60 | NNW | NE | Sleet | — | — |
| 24 | | | 40 | 44 | 38 | 30.23 | .18 | 61 | 62 | NE | W | Fine | — | — |
| 25 | | | 44 | 46 | 43 | 29.98 | 29.88 | 62 | 65 | WSW | W | Sleet | Cloudy | Cloudy |
| 26 | ○ | | 45 | 48 | 41 | .68 | .55 | 65 | 63 | W | W | Cloudy | Fine | Fine |
| 27 | | | 46 | 49 | 39 | .04 | .28 | 60 | 57 | WSW | WNW | Rain | — | — |
| 28 | | .35 | 44 | 48 | 38 | .22 | .20 | 58 | 60 | SW | W | Rain | Sho'ry | — |
| March 1 | | | 43 | 48 | 35 | .37 | .55 | 61 | 61 | W | NW | Fine | Fine | — |
| 2 | | | 39 | 46 | 39 | .69 | .80 | 61 | 62 | WNW | NW | — | — | Cloudy |
| 3 | | .18 | 42 | 48 | 43 | .59 | .51 | 62 | 66 | WSW | WSW | Cloudy | Cloudy | Rain |
| 4 | | .17 | 48 | 55 | 47 | .49 | .55 | 68 | 69 | WSW | WSW | Rain | — | — |
| 5 | | | 51 | 53 | 48 | .60 | .79 | 69 | 68 | W | WSW | Cloudy | Fine | Fine |
| 6 | ☾ | | 50 | 54 | 45 | .64 | .39 | 68 | 70 | SSW | SW | Fine | Cloudy | Cloudy |
| 7 | | | 50 | 52 | 39 | .30 | .27 | 69 | 65 | W | NW | Cloudy | Sho'ry | — |
| 8 | | | 43 | 47 | 40 | .48 | .70 | 63 | 62 | WNW | SW | — | Fine | Foggy |
| 9 | | | 50 | 51 | 38 | .59 | .38 | 61 | 61 | SW | S | — | Sleet | Cloudy |
| 10 | | .17 | 41 | 45 | 37 | .44 | .55 | 63 | 63 | W | W | — | Fine | — |
| 11 | | | 43 | 46 | 35 | .77 | .83 | 64 | 66 | W | SSW | Fine | — | — |
| 12 | | | 40 | 47 | 43 | .65 | .60 | 67 | 65 | SSW | WNW | Rain | Rain | — |
| 13 | | | 46 | 51 | 42 | .78 | .32 | 64 | 64 | SW | SW | Fine | Cloudy | Sleet |
| 14 | ● | | 47 | 50 | 40 | .50 | .44 | 62 | 62 | WSW | SW | Sho'ry | Sho'ry | — |
| 15 | | .66 | 48 | 50 | 42 | .55 | .60 | 60 | 61 | WSW | WSW | Fine | — | Fine |
| 16 | | | 48 | 55 | 47 | .63 | .38 | 63 | 66 | WSW | WNW | Rain | Cloudy | Cloudy |
| 17 | | | 51 | 54 | 53 | .54 | .67 | 67 | 68 | WSW | W | Sho'ry | Cloudy | Fine |
| 18 | | | 58 | 59 | 40 | .72 | .85 | 65 | 63 | WNW | WNW | Fine | — | — |
| 19 | | | 45 | 49 | 43 | 30.02 | 30.07 | 58 | 58 | NW | N | — | — | — |

The quantity of Rain fallen, in February, was 1 inch and 13,100ths.

